# Semi-Annual Call of the Western New York Dental Association

**Published:** 1864-05

**Authors:** B. T. Whitney, Geo. W. Field

**Affiliations:** President; Sec’y. Rochester


### SEMI-ANNUAL CALL OF THE WESTERN NEW YORK DENTAL ASSOCIATION.

ROCHESTER, March 21, 1864.

The semi-annual meeting of this Association will be held in Canandaigua on the 3d day of May, 1864. Members are particularly requested to be present, and all others who are interested in the advancement of Dentistry as a Profession.

The subjects for discussion are as follows:

Management of Deciduous Teeth. Extraction of Teeth. Hæmorrhage after extraction. Anæsthetics. Discoloration of Teeth and its removal. Treatment of sensitive dentine. Treatment of alveolar abscess. Filling teeth. Best Materials. Methods of operating in particular cases. Mechanical Dentistry, including best method of taking impressions.

Dr. B. T. WHITNEY, *President.*

GEO. W. FIELD, *Sec'y.*

